# An evaluation of the sonoporation potential of low-boiling point phase-change ultrasound contrast agents in vitro

**DOI:** 10.1186/s40349-017-0085-z

**Published:** 2017-01-24

**Authors:** Samantha M. Fix, Anthony Novell, Yeoheung Yun, Paul A. Dayton, Christopher B. Arena

**Affiliations:** 1 Eshelman School of Pharmacy, University of North Carolina Chapel Hill, Chapel Hill, NC USA; 2 Joint Department of Biomedical Engineering, University of North Carolina Chapel Hill and North Carolina State University, Chapel Hill, NC USA; 3 FIT BEST Laboratory, Chemical, Biological and Bioengineering Department, North Carolina A&T State University, Greensboro, NC USA; 4 Laboratory for Therapeutic Directed Energy, Department of Physics, Elon University, Elon, NC USA

**Keywords:** Sonoporation, Ultrasound, Drug delivery, Acoustic droplet vaporization, Nanodroplet

## Abstract

**Background:**

Phase-change ultrasound contrast agents (PCCAs) offer a solution to the inherent limitations associated with using microbubbles for sonoporation; they are characterized by prolonged circulation lifetimes, and their nanometer-scale sizes may allow for passive accumulation in solid tumors. As a first step towards the goal of extravascular cell permeabilization, we aim to characterize the sonoporation potential of a low-boiling point formulation of PCCAs in vitro.

**Methods:**

Parameters to induce acoustic droplet vaporization and subsequent microbubble cavitation were optimized in vitro using high-speed optical microscopy. Sonoporation of pancreatic cancer cells in suspension was then characterized at a range of pressures (125–600 kPa) and pulse lengths (5–50 cycles) using propidium iodide as an indicator molecule.

**Results:**

We achieved sonoporation efficiencies ranging from 8 ± 1% to 36 ± 4% (percent of viable cells), as evidenced by flow cytometry. Increasing sonoporation efficiency trended with increasing pulse length and peak negative pressure.

**Conclusions:**

We conclude that PCCAs can be used to induce the sonoporation of cells in vitro, and our results warrant further investigation into the use of PCCAs as extravascular sonoporation agents in vivo.

**Electronic supplementary material:**

The online version of this article (doi:10.1186/s40349-017-0085-z) contains supplementary material, which is available to authorized users.

## Background

Sonoporation refers to the process by which ultrasound-stimulated microbubbles are used to permeabilize cell membranes and enhance the intracellular accumulation of drugs, genes, or indicator dyes [1, 2]. This holds potential as a physical targeting method to drive drug delivery non-invasively and with high spatial specificity. However, inherent limitations associated with microbubble contrast agents used for previous in vitro investigations [3–7] must be overcome to enable in vivo translation and subsequent widespread utility of this technique.

First, microbubbles are relatively large (1–10 μm) and therefore cannot escape the vasculature following intravenous administration [8]. This can be useful for some applications including drug or gene delivery to vascular endothelial cells [9] and ultrasound-mediated disruption of endothelial tight junctions to open the blood-brain barrier [10, 11]. However, extravascular sonoporation for the purpose of improved drug or gene delivery within a target tissue is not feasible. Second, microbubbles have limited persistence in circulation (<10 min) [8]. This necessitates continuous infusion or repeat bolus injections in situations where repeat or long duration treatment is required.

Phase-change ultrasound contrast agents (PCCAs) are nanometer scale, liquid-filled droplets that can be vaporized into microbubbles when subjected to ultrasound of sufficient amplitude through a process termed acoustic droplet vaporization (ADV). These agents are characterized by longer circulation half-lives than similarly formulated microbubbles [12, 13], and their nanometer-scale size distributions may allow for passive accumulation in leaky tumors via the enhanced permeability and retention (EPR) effect [13, 14]. Furthermore, since PCCAs are nearly invisible to ultrasound in their liquid state, high concentrations can be used without the shielding effect characteristic of high microbubble concentrations. PCCA-derived microbubbles destroyed in one acoustic pulse may be replenished through subsequent vaporization events, thereby allowing for the sustained generation of cavitation energy and enhanced sonoporation [15]. PCCAs therefore offer a solution to the major limitations previously given for microbubble-mediated sonoporation and hold the potential for extravascular sonoporation in vivo.

PCCA formulations are commonly filled with perfluorocarbons with boiling points near body temperature, such as dodecafluoropentane (DDFP, bp = 29 °C), and a few laboratories have demonstrated the sonoporation potential of such agents in vitro [15–18]. While these initial studies show promise, the high negative pressures required to vaporize nanoscale DDFP-filled PCCAs (3–6 MPa [14, 15]) may cause unwanted bioeffects such as heating or cell lysis in an in vivo setting. Our laboratory has developed a class of low-boiling point PCCAs filled with octofluoropropane (OFP, bp = −36.7 °C), which are characterized by far lower pressure requirements for vaporization when compared to DDFP-filled PCCAs (~20× lower). Therefore, we hypothesize that our formulation will offer greater control over the bioeffects caused by ADV and subsequent microbubble cavitation. The primary objective of this study is to characterize the sonoporation potential of these low-boiling point PCCAs in vitro.

The precise mechanisms involved in PCCA-mediated sonoporation remain unknown, and likely depend on a number of factors including the contrast agent formulation, specific acoustic parameters (frequency, peak negative pressure [3], duty cycle, etc.), and non-acoustic parameters (microbubble size and bubble-to-cell distance [19], cell culture conditions, size of sonoporation indicator [3], etc.). It is conceivable that PCCA-induced sonoporation is driven by the same mechanisms that mediate microbubble sonoporation, with membrane permeabilization being a product of microbubble cavitation following ADV. However, the rapid expansion of an individual droplet as it phase-converts into a microbubble may itself influence cell permeability. A secondary objective of this study is to determine if the vaporization event of low-boiling point PCCAs contributes to sonoporation and/or effects cell viability.

## Methods

### Fabrication and characterization of phase-change ultrasound contrast agents

Low-boiling point PCCAs containing liquid octafluoropropane (OFP, boiling point −36.7 °C) were generated as described elsewhere [20]. First, lipid-shelled, OFP-filled microbubbles were prepared. Briefly, 90 mol% 1,2-distearoyl-*sn*-glycero-3-phosphocholine (DSPC) and 10 mol% 1,2-distearoyl-*sn*-glycero-3-phosphoethanolamine-*N*-methoxy(polyethylene-glycol)-2000 (DSPE-PEG2000) (Avanti Polar Lipids, Alabaster, AL, USA) were combined and dissolved in a phosphate-buffered saline (PBS)-based solution containing 15% propylene glycol (*v*/*v*) and 5% glycerol (*v*/*v*) for a final lipid concentration of 1.0 mg/mL. This lipid solution (1.5 mL) was aliquoted into 3.0 mL glass vials and the headspace air was exchanged with OFP gas (FluoroMed, Round Rock, TX, USA). Finally, microbubbles were generated by vigorous shaking of the lipid vials using a VialMix (Bristol-Myers-Squibb, New York, NY, USA).

The OFP microbubbles were condensed into liquid-filled nanodroplets (i.e., PCCAs) [20]. Microbubble vials were cooled in an isopropanol/CO_2_ bath maintained between −10 and −13 °C. Simultaneously, the headspace pressure of the vials was gradually increased through the addition of excess OFP gas until microbubble condensation was observed. Phase transition is visually apparent, as the initially opaque microbubble solution turns translucent when condensed into liquid-filled particles.

**Fig. 1:**
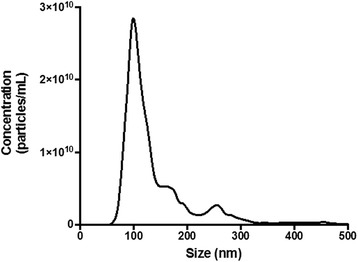
Nanosight results for OFP-filled PCCAs (*N* = 3 vials). The mean particle size (±SD) was found to be 140 ± 10 nm and the average concentration (±SD) was 1.7 ± 0.1 × 10^12^ particles/mL

The size distribution and concentration of the PCCAs were characterized using a NanoSight NS500 (Malvern Instruments, Westborough, MA, USA) capable of detecting nanoparticles between 50 and 2000 nm. PCCAs were diluted 3000-fold in HPLC-grade, 20 nm filtered water. Four, 30-s recordings were captured per sample to calculate an average size distribution and concentration for each sample. This procedure was repeated in triplicate for three separate vials of PCCAs and averaged to get a representative size distribution and concentration. The particles were characterized by a polydisperse size distribution, as in Fig. 1, with a mean size of 143 ± 13 nm and concentration of 1.7 ± 0.1 × 10^12^ particles/mL (see Additional file 1 for error estimation).

### Visualization of PCCA vaporization and secondary microbubble affects using optical microscopy and high-speed photography

High-speed optical microscopy was used to detect PCCA vaporization following ultrasound stimulation using a previously described experimental setup [21, 22]. Briefly, an inverted microscope with a 100× water immersion objective (Olympus IX71, Center Valley, PA, USA) was interfaced with a high-speed camera (FastCam SA1.1, Photron USA, Inc., San Diego, CA, USA). The objective was submerged in a temperature-controlled water bath fixed on top of the microscope. The water bath was filled with degassed water and held at 37 °C. A solution of PCCAs diluted in PBS (6.7% *v*/*v*) was injected into a microcellulose tube (200-μm inner diameter) (Spectrum Labs, Inc., Rancho Dominguez, CA, USA) positioned over the optical focus. This injection was followed by a brief waiting period to allow the flowing particles to become nearly stationary. This enabled clear visualization of vaporization events as images become blurred when particles are flowing.

A 1.0-MHz spherically focused piston transducer (diameter = 19 mm, focal distance = 38 mm, IL0106HP, Valpey Fisher Corp., Hopkinton, MA, USA) was submerged in the water bath and positioned such that the acoustic focus was aligned with the microcellulose tube at the optical focus as described previously [22]. Briefly, a calibrated needle hydrophone (HNA-0400, Onda Corp., Sunnyvale, CA, USA) was aligned with the microscope focus and used to subsequently align the focus of the transducer to that location. The hydrophone was then used to calibrate the pressure output of the transducer at various excitation voltages. The transducer was excited with sinusoidal pulses generated with an arbitrary waveform generator (AFG3021C, Tektronix, Inc., Beaverton, OR, USA) and amplified approximately 60 dB with a power amplifier (A500, ENI, Rochester, NY, USA). Following calibration, the hydrophone was replaced with a microcellulose tube, which was aligned with the microscope focus. In this way, we ensured that the plane of the tube visible in the optical focus was subjected to the calibrated acoustic pressures aligned to that location.

PCCAs flowing through the microcellulose tube were exposed to acoustic pulses with lengths of 5, 10, 20, and 50 cycles and peak negative pressures of 125, 300, 600, 1000, and 2000 kPa to observe the effect of pulse length and pressure on PCCA vaporization. In subsequent experiments, pre-vaporized PCCAs were stimulated with a second identical acoustic pulse to observe how ultrasound affected the generated microbubbles.

A synchronization pulse from the waveform generator was used to trigger the high-speed camera. Video recordings were set to begin just before the manually triggered ultrasound pulse such that vaporization or microbubble manipulations would be recorded in their entirety. A frame rate of 500 frames per second was employed. Images and videos were stored on a computer using proprietary camera software (PFV; Photron USA, Inc., San Diego, CA, USA) and analyzed using ImageJ (NIH, Bethesda, MD, USA).

### Detection of cavitation signals following PCCA vaporization

Similar to the high-speed microscopy experiments, PCCA solutions were perfused through a microcellulose tube (200 μL/min) aligned with the focus of a 1.0-MHz piston transducer. The transducer was calibrated at the focus using a needle hydrophone, and PCCAs were activated with sinusoidal ultrasound pulses using a pulse repetition frequency (PRF) of 5.0 Hz, peak negative pressures ranging from 125 to 2000 kPa, and pulse lengths between 5 and 50 cycles. Three concentrations of PCCAs were tested: 0.067, 0.67, and 6.7% (*v*/*v*) in PBS. All conditions and concentrations were tested in triplicate using three independent vials of PCCAs. Control trials with a water-filled tube were used as a reference to estimate stable and inertial cavitation generated by the vaporized PCCAs.

To detect cavitation signals, a separate, spherically focused receive transducer (7.5 MHz center frequency, diameter = 19 mm, focal distance = 50 mm) (V321, Panametrics, Inc., Waltham, MA, USA) was positioned perpendicular to the transmit transducer such that the microcellulose tube was aligned with both transducer foci. Signals from the receive transducer were acquired using a 14-bit analog to digital conversion (ADC) card with a sampling frequency of 100 MHz (PDA14, Signatec, Corona, CA, USA) installed in a computer (Dell, Round Rock, TX, USA) running a custom acquisition program (LabVIEW, National Instruments Corp., Austin, TX, USA). A total of 50 individual signals were captured for each combination of pressure, pulse length, and PCCA concentration. These signals were saved and post-processed using MATLAB (MathWorks Inc., Natick, MA, USA).

A custom MATLAB script was developed to quantify the energy of stable and inertial cavitation generated for each condition. First, a window from 50–110 μs referenced to the beginning of the acoustic pulse was applied to select the signal emitted by the PCCAs. The 50 individual RF signals from each exposure condition were converted into the frequency domain. Detection of the second harmonic component was used to estimate the stable cavitation level by filtering the data from 1.8 to 2.2 MHz (Butterworth filter, order 3). The broadband signal resulting from inertial cavitation was detected by filtering the signals from 5.25 to 7.75 MHz (Butterworth filter, order 3) and by simultaneously excluding the harmonic components at 6 and 7 MHz. Finally, energies of these stable and inertial cavitation signals were calculated, averaged among the 50 individual signals for each condition, and normalized by the energy calculated for a water-filled tube exposed to the same acoustic conditions. This procedure was repeated for three independent vials of PCCAs. The average, normalized cavitation energies are reported with the inter-vial standard deviation.

### Cell culture

Human pancreatic adenocarcinoma cells (PANC-1) were purchased from American Type Culture Collection (ATCC, VA, USA) and cultured in Dulbecco’s Modified Eagle’s Medium (DMEM) supplemented with 10% fetal bovine serum (FBS) and 1% penicillin-streptomycin (Sigma-Aldrich Co., MO, USA) at 37 °C and 5% CO_2_ atmosphere. For all experiments, cells between passages 5 and 24 were used. Cells were harvested using trypsin-EDTA (Sigma-Aldrich Co., MO, USA) and counted using a hemocytometer for use in sonoporation and viability experiments.

### Sonoporation of cells in suspension

**Fig. 2:**
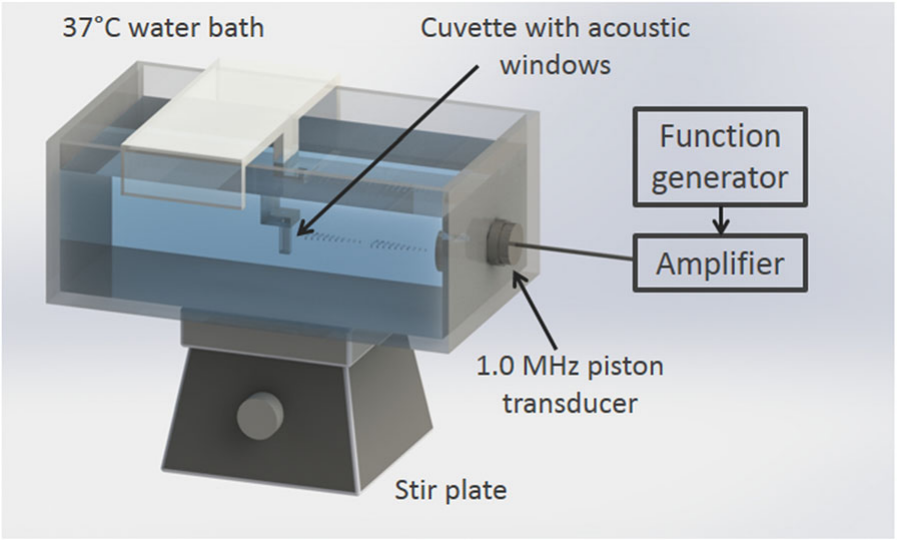
Setup designed for the sonoporation of cells in suspension with PCCAs

PANC-1 cells (1.0 × 10^6^ cells) were suspended in serum-free DMEM containing PCCAs (8.5 × 10^8^ particles) and propidium iodide (PI, 30 μM) (Sigma-Aldrich Co., MO, USA) for a final volume of 1.5 mL. PI was used as a sonoporation indicator as it is impermeable to intact cell membranes. The cell suspension was added to a custom plastic cuvette with nearly acoustically transparent windows made of 20-μm thick polyolefin film (Rajashrink, Roissy, France) as previously described by Escoffre et al. [23]. The cuvette was then held in a 37 °C degassed water bath with constant magnetic stirring and positioned 5 cm in front of the transducer for sonoporation treatment, as shown in Fig. 2.

To generate ultrasound pulses, a 1.0-MHz unfocused piston transducer (diameter = 2.54 cm, IL0108HP, Valpey Fisher Corp., Hopkinton, MA, USA) was excited by a sinusoidal arbitrary function generator signal (AFG3021C, Tektronix, Inc., Beaverton, OR, USA) amplified approximately 55 dB by an RF power amplifier (3100LA, ENI, Rochester, NY, USA). The pressure output of the transducer at various excitation voltages was characterized using a calibrated needle hydrophone placed 5 cm in front of the transducer, matching the distance of the cuvette in sonoporation experiments. The cell suspensions were insonified for 30 s with peak negative pressures of 125, 300, or 600 kPa, pulse lengths of 5, 10, 20, or 50 cycles, and a constant PRF of 5.0 kHz, as summarized in Table 1. As controls, cells underwent (1) sham treatment (without PCCAs or ultrasound exposure) and (2) ultrasound-only treatment (without PCCAs) using the highest energy condition—600 kPa and 50 cycles.

**Table 1:** Experimental and control conditions for sonoportion

Conditions	PCCAs (Y/N)	Cycles (#)	PRF (kHz)	Pressure (kPa)
1–4	Y	5, 10, 20, 50	5	125
5–8	Y	5, 10, 20, 50	5	300
9–12	Y	5, 10, 20, 50	5	600
US only control	N	50	5	600
Sham control	N	NA	NA	NA

Post-treatment, cells were transferred to plastic tubes and incubated at 37 °C for at least 15 min to ensure membrane resealing processes were completed prior to further manipulation of the cells [24]. Subsequently, the viability stain calcein-AM (0.8 μM) (Thermo Fisher Scientific Inc., MA, USA) was added and the cells were allowed to incubate for at least an additional 30 min at 37 °C. Cells were filtered through a 44-μm nylon mesh (Component Supply Co., FL, USA) before being analyzed by flow cytometry. Cells showing both PI uptake and calcein-AM cleavage by flow cytometry were considered to be successfully sonoporated. These experiments were repeated in triplicate on independent days. All sonoporation conditions were also performed in triplicate without the addition of dye to monitor changes in autofluorescence due to treatment.

### Assessment of sonoporation efficiency by flow cytometry

Flow cytometry was used to quantify the number of sonoporated cells for each treatment group, i.e., those cells displaying both PI uptake (permeabilization) and calcein-AM cleavage (viability). An LSRFortessa cytometer equipped with 561- and 488-nm excitation lasers (Becton Dickinson, Franklin Lakes, NJ, USA) was used for acquisition, and 30,000 events were recorded for each sonoporation treatment. For further details regarding acquisition settings, please see Additional file 1: Table S2.

The gating strategy employed to isolate sonoporated cells is described in full in Additional file 1: Information section and displayed in Additional file 2: Figure S1. Briefly, singlet cells were isolated from debris and doublet cells through initial gating steps. The viability of the chosen cell population was then confirmed by calcein fluorescence. Curly quadrant gates were applied to the calcein vs. PI fluorescence dot plots, with thresholds determined such that unstained control cells would be classified as both calcein and PI negative. The percent of cells in quadrant two (calcein and PI positive) was taken to be the sonoporation efficiency (i.e., percent of viable cells that were sonoporated). All data analysis was performed using FlowJo Data Analysis Software (FlowJo, LLC., Ashland, OR, USA).

### Assessing viability post-sonoporation treatment

Through our flow cytometry experiments, we found that dead cells and cellular debris were characterized by elevated autofluorescence in the calcein (viability) channel (data not shown). Therefore, we were unable to accurately quantify cell viability based on the flow cytometry results alone. As such, we performed an additional cell viability assay. Cells suspended in serum-supplemented DMEM were subjected to the sonoporation protocol as described above without the addition of PI or calcein-AM. Following treatment, 1.0 × 10^5^ cells per treatment group were transferred to 24-well plates and allowed to incubate for 24 h at 37 °C and 5% CO_2_ atmosphere. Subsequently, cell viability was assessed using a resazurin-based toxicology assay according to the manufacturer’s protocol (Sigma-Aldrich Co., MO, USA).

Briefly, a volume of resazurin dye equal to 10% of the culture media was added to the cells and allowed to incubate for 3 h. A 200-μL sample from each culture well was then transferred to a 96-well plate for analysis. The fluorescence increase at 590 nm (F_590_) due to reduction of the resazurin dye by viable cells was detected using a plate reader (Synergy 2, BioTek Instrument, Inc., Winooski, VT, USA) with excitation and emission filters of 530/25 nm and 590/35 nm, respectively. The fluorescence intensity of a blank sample containing complete media but no cells was subtracted from that of each sample. Cell viability was then calculated as the percent resazurin reduction of the sham control. Viability experiments were repeated in triplicate on independent days.

### Statistical analyses

All statistical analyses were performed in GraphPad Prism 7 (GraphPad Software, Inc., La Jolla, CA, USA), and data are presented as average ± standard deviation throughout this work. Sonoporation efficiencies and cell viabilities were compared among treatment groups using one-way ANOVA followed by Dunnett’s multiple comparison testing on significant results. Each treatment group was compared to the sham control, and *p* values of <0.05 were considered statistically significant. Pearson correlation coefficients (*r*) were computed in GraphPad Prism 7 to analyze the correlation between (1) sonoporation efficiency and stable cavitation and (2) sonoporation efficiency and inertial cavitation. Correlations were considered statistically significant if the two-tailed *p* values were <0.05.

## Results

### Detection of PCCA vaporization and subsequent cavitation signals

**Fig. 3:**
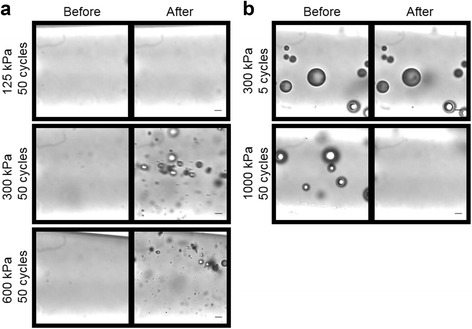
Observation of PCCA vaporization and secondary microbubble effects using high-speed photography. Representative photos are displayed of PCCAs or resultant microbubbles before and after ultrasound stimulation (1.0 MHz center frequency). **a** The nanoscale, liquid-filled PCCAs are difficult to observe before vaporization. A peak negative pressure of 125 kPa is not sufficient to vaporize the PCCAs (*top*). With a peak negative pressure of 300 kPa, efficient vaporization of the PCCAs into microbubbles is observed (*bottom*). **b** Secondary effects are observed when generated microbubbles are subjected to a second acoustic pulse. At 300 kPa and 5 cycles, the second acoustic pulse appears to have no affect on the generated microbubbles (*top*). With high acoustic energies, complete microbubble destruction is observed (*bottom*). *Scale bar* = 10 μm. **a** Vaporization **b** Secondary effects

Through optical high-speed microscopy and the detection of cavitation signals, we investigated the effect of acoustic pulse length and peak negative pressure on PCCA vaporization (a.k.a. acoustic droplet vaporization (ADV)) and the behavior of resultant microbubbles. At a frequency of 1.0 MHz, we found that our PCCAs undergo ADV at and above peak negative pressures of 300 kPa but never at or below 125 kPa, regardless of pulse length. This is consistent with previous reports demonstrating that ADV is a pressure-dependent, threshold phenomenon that is independent of pulse length when short, microsecond pulses are used [25, 26]. Representative photos showing PCCAs before and after ultrasound stimulation above and below the activation threshold are displayed in Fig. 3a. Note that 300 kPa does not represent an absolute pressure threshold for vaporization; rather, we conclude that the vaporization threshold is between 125 and 300 kPa under the conditions studied.

While pulse length did not effect whether or not vaporization would occur, it did influence the behavior of resultant microbubbles. When generated microbubbles were stimulated with a second ultrasound pulse, microbubble destruction occurred on a continuum. No destruction was observed with low-pressure pulses (300 kPa) and complete destruction of all microbubbles in the field of view occurred with long pulses (20 and 50 cycles) at high pressure (1000 and 2000 kPa) (Fig. 3b).

Microbubble sizes were estimated from the captured images. Microbubbles generated from ADV at 300 kPa were polydisperse and ranged in size between 2 and 10 μm. When the peak negative pressure was increased to 600 kPa and above, generated microbubbles were observed in the 1- to 10-μm range; however, we note an increase in the number of small (~1 μm) microbubbles present. This is consistent with previous reports from our laboratory detailing the dependence of generated microbubble size on various acoustic parameters, including peak negative pressure [27]. The shift towards smaller resultant microbubbles with increased pressure is due to the inverse relationship between vaporization threshold and PCCA size [27, 28]. When generated microbubbles were allowed to rest before being subjected to a second acoustic pulse (Fig. 3b), we noticed microbubble sizes shift to be larger (approximately 3–20 μm). This is likely due to coalescence of the generated microbubbles.

**Fig. 4:**
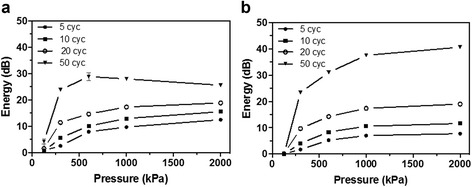
Quantification of the **a** stable and **b** inertial cavitation energy generated by PCCAs subjected to ultrasound of various peak negative pressures and pulse lengths. Note: while error bars (SD) are plotted, they are not visible on all data points due to their small size. **a** Stable Cavitation Detection **b** Inertial Caviatation Detection

The generation of stable and inertial cavitation signals depended on peak negative pressure and pulse length. Very little stable and no inertial cavitation was observed at pressures of 125 kPa regardless of pulse length; the slight stable cavitation may be due to oscillations of microbubbles that arose from spontaneous vaporization. Cavitation energy was observed from 300 to 2000 kPa, with very little cavitation achieved with peak negative pressures of 300 kPa and short (5 and 10 cycle) pulse lengths (Fig. 4). The amount of stable cavitation produced reached a plateau between 24 and 29 dB for 50 cycle pulses with pressures between 300 and 2000 kPa. Alternatively, inertial cavitation continued to increase with increasing pressure. Interestingly, the concentration of PCCAs did not significantly influence the amount of stable or inertial cavitation detected (data not shown), and obtained graphs for all tested concentrations were nearly identical to the one presented in Fig. 4 for 0.67% (*v*/*v*) PCCAs in PBS.

### Sonoporation efficiency

Flow cytometry was used to analyze the effect of acoustic pressure and pulse length on PCCA-facilitated PI uptake through sonoporation. Dead cells were discarded from the analysis through an initial gating step to remove cellular debris (Additional file 2: Figure S1). The viability of remaining cells was confirmed using calcein-AM staining. While the viability of gated cells was near 100% for all conditions, the percentage of cellular debris was observed to increase with increasing acoustic energy, implying elevated cell death.

**Fig. 5:**
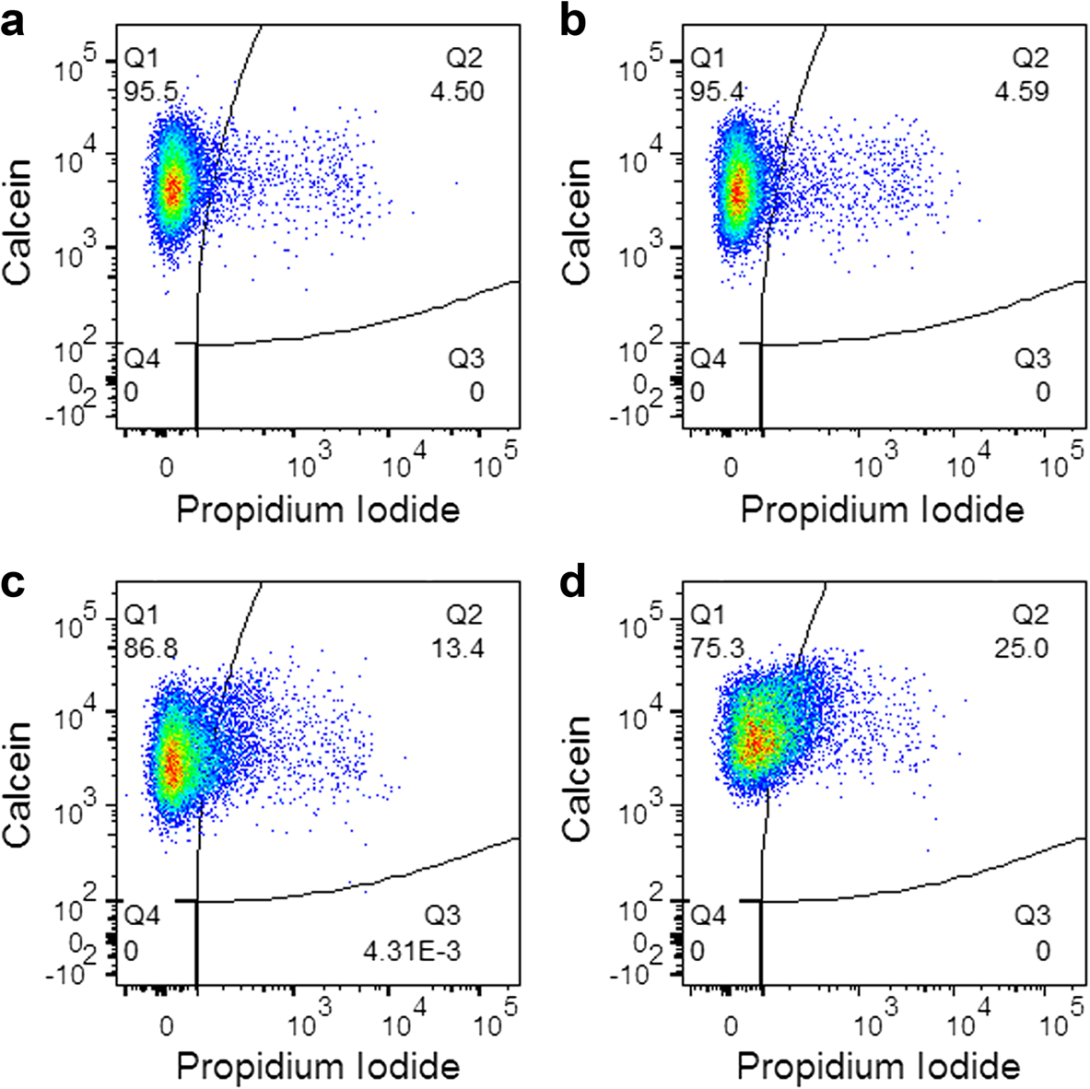
Representative flow cytometry dot plots used to quantify sonoporation efficiency. Cells were classified as sonoporated if they showed calcein fluorescence (viability) and uptake propidium iodide (membrane permeability). These cells appear in quadrant 2 (Q2) in the dot plots. **a** The small percentage of cells that appear in Q2 for the sham treatment group was defined as the false positive rate and was subtracted from the percent of cells in Q2 for all treatment groups. **b** Ultrasound exposure below the PCCA activation threshold did not result in sonoporation. **c**, **d** Sonoporation is observed above the PCCA activation threshold and increases with increasing pressure. **a** Sham Control **b** 125 kPa - 20 cycles **c** 300 kPa - 20 cycles **d** 600 kPa - 20 cycles

Sonoporation efficiency, the percent of viable cells displaying PI fluorescence, was quantified as the percent of cells in quadrant 2, as shown in Fig. 5. A small percentage of cells (2–4.5%) appeared in quadrant two for the sham control, likely due to the prolonged exposure of cells to PI. This was defined as the false positive rate and was subtracted from the sonoporation efficiency of all other treatment groups. The autofluorescence analysis demonstrated slight spreading of unstained cell populations along the PI axis due to ultrasound treatment with PCCAs (Additional file 3: Figure S2). The average percent of cells classified as PI positive due to autofluorescence never exceeded 2%, but these values were subtracted from the final sonoporation efficiencies of all groups.

**Fig. 6:**
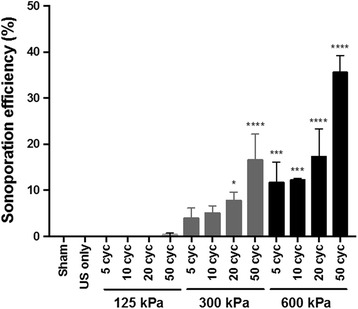
Sonoporation efficiency of PANC-1 cells at various acoustic pressures and pulse lengths. As expected, we do not observe sonoporation below the vaporization threshold of the PCCAs (i.e., at 125 kPa) or when cells are insonified in the absence of PCCAs (US alone). One-way ANOVA was used followed by Dunnett’s multiple comparisons test compare each treatment to the sham control. *p ≤ 0.05, ***p ≤ 0.001, ****p ≤ 0.0001

Statistically significant elevation in PI uptake was observed at 300 kPa with pulse lengths of 20 and 50 cycles and at 600 kPa with 5–50 cycle pulse lengths compared to the sham control. Sonoporation efficiency increased with peak negative pressure and pulse length, reaching a maximum of 36 ± 4% at 600 kPa and 50 cycles (Fig. 6). As expected, we did not observe sonoporation below the vaporization threshold of the PCCAs (i.e., at 125 kPa) or when cells were insonified in the absence of PCCAs.

### Cell viability 24 h post-treatment

**Fig. 7:**
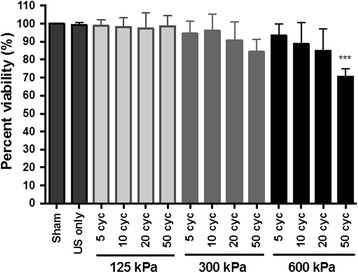
Cell viability 24 h post-sonoporation treatment. Here, we observe decreasing cell viability with increasing pulse length and pressure. As expected, ultrasound exposure in the absence of PCCAs does not affect cell viability. Furthermore, the PCCAs themselves did not have a toxic effect on cells as evidenced by the high viability in treatment groups below the PCCA activation threshold (i.e., 125 kPa groups). One-way ANOVA was used followed by Dunnett’s multiple comparisons test to compare each treatment to the sham control. ***p ≤ 0.001

To test the effect of sonoporation treatment on cell viability, cells were treated using a protocol identical to that employed for sonoporation but without the addition of PI or calcein-AM. Twenty-four hours post-treatment, viability was assessed using a resazurin-based metabolic assay. Ultrasound exposure in the absence of PCCAs did not affect cell viability. Furthermore, the PCCAs themselves did not have a toxic effect on cells as evidenced by the high viability in treatment groups below the PCCA activation threshold (i.e., 125 kPa treatment groups). We did observe decreasing cell viability with increasing cycle number and pressure above the activation threshold. In general, fairly high viability was recorded for those cells treated with 300 kPa ultrasound of various pulse lengths (84 ± 7%–94 ± 7% viability) and cells treated with 600 kPa ultrasound with pulse lengths between 5 and 20 cycles (85 ± 12%–93 ± 6%) (Fig. 7). A statistically significant drop in viability (70 ± 5%) was observed in cells treated with 600 kPa and 50 cycles compared to sham treated cells.

## Discussion

### Acoustic or temperature-induced droplet vaporization can be achieved without membrane perforation or impaired cell viability

Our PCCAs are comprised of a very low-boiling point PFC and undergo some spontaneous vaporization when incubated at 37 °C. Therefore, cells incubated with PCCAs and exposed to ultrasound below the activation threshold (i.e., at 125 kPa) felt the effects of temperature-induced vaporization alone. The membrane permeability and viability of cells treated in this way was unaltered. Additionally, cells treated with PCCAs at 300 kPa with pulse lengths of 5 or 10 cycles demonstrated insignificant sonoporation efficiencies and no change in cell viability. These cells were exposed to acoustic droplet vaporization but minimal cavitation of the resultant microbubbles. These data indicate that vaporization events do not affect cellular membrane permeability or cause any detrimental cellular bioeffects.

This is in contrast to the bioeffects observed following the vaporization of micron-sized, DDFP-filled PCCAs used for vascular occlusion. Seda, et al. have demonstrated that vaporization of DDFP-filled droplets results in extensive cell death even when using acoustic parameters designed to minimize secondary mechanical effects from the resultant bubbles [29]. Differences in experimental setups (cells treated in adherent culture vs. in suspension) and size distributions of PCCAs (1.6 ± 0.5 μm vs. 143 ± 13 nm mean size) make it difficult to directly compare these results. However, the difference in severity of bioeffects observed is likely due to the difference in pressure required to vaporize the PCCAs. Rarefactional pressures of at least 6 MPa were required for vaporization of DDFP-filled PCCAs, while 300 kPa was sufficient for vaporization of our PCCAs. By using a highly volatile formulation with lower pressure requirements for ADV, we can safely induce vaporization without immediately and irreparably damaging surrounding cells.

### PCCA-induced sonoporation is correlated with stable and inertial cavitation

**Fig. 8:**
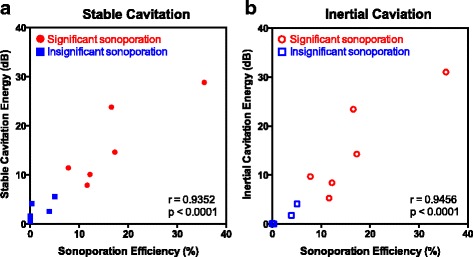
We observe a strong, positive correlation between sonoporation efficiency and **a** stable cavitation and **b** inertial cavitation. The Pearson correlation coefficients for sonoporation efficiency vs. stable and inertial cavitation are 0.9352 and 0.9456, respectively, and both correlations are statistically significant with *p* values <0.0001. Data points corresponding to statistically significant sonoporation efficiencies (compared to sham control) are shown in *red circles*, while data points corresponding to statistically insignificant sonoporation efficiencies are shown in *blue squares*. **a** Stable Cavitation **b** Inertial Caviation

Sonoporation efficiency was found to be significantly and positively correlated with both stable (*r* = 0.9352, *p* < 0.0001) and inertial (*r* = 0.9456, *p* < 0.0001) cavitation (Fig. 8). While it is difficult to ascertain a cavitation threshold for sonoporation from these data, we note that all statistically significant sonoporation treatments were associated with stable cavitation energies greater than 7.9 dB and inertial cavitation energies greater than 5.2 dB. This study was not designed to elucidate the mechanisms driving PCCA-mediated sonoporation, but our data suggest that the mechanical effects due to microbubble-ultrasound interactions are necessary for significant sonoporation. Therefore, it is likely that the same mechanisms that drive conventional microbubble-mediated sonoporation also drive PCCA-mediated sonoporation.

The peak sonoporation efficiency we achieved (36%) is similar to what has previously been reported for microbubble sonoporation (28–39% efficiency) [6, 30, 31], albeit with lower cell viability (70% viability for PCCA sonoporation vs. 90–96% viability for MB sonoporation [6, 30, 31]). However, these microbubble sonoporation studies employ unique strategies to increase sonoporation efficiency and minimize cell death, making it difficult to make direct comparisons. For example, McLaughlan et al. achieved their highest viable sonoporation using a combination of (1) targeted microbubbles that increase cell-microbubble interactions and (2) chirp frequency excitation to maximize the response of their polydisperse microbubbles [6]. Song et al. found that using monodisperse 2.0-μm microbubbles resulted in the highest sonoporation and viability after a single ultrasound treatment [31]. We believe that with further optimization of our PCCA-mediated sonoporation methods, we will be able to match the sonoporation efficiencies and viabilities achieved with microbubbles. Future studies will be designed to apply the aforementioned techniques developed by the microbubble sonoporation community to PCCA-mediated sonoporation.

## Conclusions

In conclusion, our data show that low-boiling point PCCAs are capable of inducing sonoporation without causing detrimental cellular bioeffects in vitro. Furthermore, the low pressure required to activate such PCCAs allows us to fine-tune the severity of cellular bioeffects simply by modifying pulse length. This provides flexibility in future applications imaginable and allows for acoustic droplet vaporization to be achieved safely and with existing diagnostic imaging hardware. Here we demonstrate the ability to cause (1) vaporization with no cellular damage—ideal for diagnostic imaging applications, (2) reversible sonoporation—desirable for therapeutic applications such as drug or gene delivery where cell death is to be avoided, or (3) irreversible sonoporation—useful in augmenting tumor killing through high-intensity focused ultrasound treatment.

A limitation of this study is that we did not control for differences in PCCA vaporization efficiency at each acoustic condition. In other words, more bubbles were likely generated using the highest energy conditions compared to the lowest energy conditions as a constant PCCA concentration was used throughout. This makes it difficult to draw conclusions about sonoporation mechanism and parameter optimization. The increases in sonoporation efficiency with increasing pressure and pulse length may have been due to (1) increased cavitation and associated mechanical effects, (2) increased concentration of generated microbubbles, or (3) a combination thereof. Future studies will be designed to quantify the vaporization efficiency of PCCAs at each acoustic condition to allow for concentration matching of generated microbubbles. Other important parameters to consider are contrast agent size distribution, ultrasound exposure duration, and center frequency. Future studies will be designed to optimize these parameters and provide a thorough comparison between the sonoporation potential of microbubbles, low-boiling point PCCAs, and high-boiling point PCCAs.

One of the main advantages of using PCCAs for reversible sonoporation compared to microbubbles is the potential for their extravasation from a tumor’s leaky vasculature. While we note that the mean size of our PCCAs is smaller than the pore sizes in many permeable tumor lines (200–1.2 μm) [32], the extravasation and accumulation of our particles in tumors has yet to be confirmed. Studies are currently ongoing towards this end. Nevertheless, our data warrant further investigation into the use of PCCAs to induce extravascular sonoporation in vivo for the purpose of enhancing local drug or gene delivery, particularly within solid tumors.

## Additional files

Additional file 1: S.1. Error estimation for PCCA size and concentration. Table S1. Error estimation for PCCA size and concentration. S.2. Details regarding flow cytometry analysis. Table S2. Detector voltages used for all flow cytometry acquisitions. (DOCX 25 kb)
Additional file 2: Figure S1. Gating hierarchy used for sonoporation detection. (TIF 114 kb)
Additional file 3: Figure S2. A) Gating hierarchy for detecting autofluorescence in treated cells. First, cells were isolated from debris using FSC-A vs. SSC-A. Second, singlet cells were isolated using FSC-A vs. FSC-H. Third, quadrant gates were drawn identical to those used for quantifying sonoporation. B) Representative dot plots demonstrating slight spreading (autofluorescence) of cells treated with ultrasound and PCCAs. (TIF 123 kb)

## Abbreviations

ADV: Acoustic droplet vaporization
DDFP: Dodecafluoropentane
OFP: Octafluoropropane
PCCA: Phase-change contrast agent
PI: Propidium iodide
US: Ultrasound
