# Supplementary material for: An evaluation of the sonoporation potential of low-boiling point phase-change ultrasound contrast agents in vitro
Source: J Ther Ultrasound. 2017 Jan 24;5:7. doi: 10.1186/s40349-017-0085-z (PMC5260003; doi:10.1186/s40349-017-0085-z)
Supplement: Additional file 1: — S.1. Error estimation for PCCA size and concentration. Table S1. Error estimation for PCCA size and concentration. S.2. Details regarding flow cytometry analysis. Table S2. Detector voltages used for all flow cytometry acquisitions. (DOCX 25 kb) [file 40349_2017_85_MOESM1_ESM.docx]

**Additional file 1**

**Supporting information**

**S.1. Error estimation for PCCA size and concentration**

Each individual PCCA sample was measured four times to give a representative size distribution and concentration, each with an associated standard deviation (intra-sample SD). This was repeated for three independent vials of PCCAs giving an overall average size and concentration with an associated standard deviation (inter-vial SD). The intra-sample SD for both size and concentration was found to be greater than the corresponding inter-vial SDs (Table S1). As such, slight variations between vials were negligible given the uncertainty associated with measurement, and vials used throughout the experiments were assumed to be identical and to be characterized by the size distribution and concentration presented. In the body of this text, average size and concentration was reported with their associated intra-sample SDs.

**Table S1:** Error estimation for PCCA size and concentration.

| Characteristic | Intra-sample SD | Inter-vial SD |
| --- | --- | --- |
| Size (nm) | ± 13 | ± 3.5 |
| Concentration (#/mL) | ± 1.2×10^11^ | ± 0.67×10^11^ |

**S.2 Details regarding flow cytometry analysis**

All flow cytometry experiments were run using an LSRFortessa cytometer equipped with 488 nm and 561 nm excitation lasers to detect calcein and propidium iodide (PI) fluorescence, respectively (Becton Dickinson, Franklin Lakes, NJ, USA). Detector voltages (gains) were kept consistent throughout the experiments and are presented in Table S2.

**Table S2:** Detector voltages used for all flow cytometry acquisitions

| Detector | Voltage (V) |
| --- | --- |
| Front scatter (FSC) | 162 |
| Side scatter (SSC) | 164 |
| Calcein | 130 |
| Propidium Iodide | 242 |

There is very little spectral overlap between calcein and PI; nevertheless, compensation matrices were calculated for each experiment. The following were used as compensation controls: (1) untreated, unstained cells, (2) untreated cells stained only with calcein-AM, and (3) ethanol-killed cells stained only with PI. Ten thousand events were recorded for each. Compensation matrices were calculated using FlowJo Data Analysis Software (Ashland, OR, USA) and were used to compensate all sonoporation data.

To quantify the number of viable sonoporated cells the following gating strategy was employed. First, cells were isolated from debris using front scatter area (FSC-A) vs. side scatter area (SSC-A) characteristics. Second, singlet cells were isolated using FSC-A vs. front scatter height (FSC-H). Third, viability of the isolated cell population was confirmed by calcein fluorescence. Finally, a curly quadrant gate was drawn on the calcein vs. PI dot plot to quantify the percent of sonoporated cells as those displaying both calcein-AM cleavage (viable) and PI uptake (permeabilized) (quadrant two (Q2)). Curly quadrant gates (those with curved arms) were employed in effort to minimize error associated with spread of intensely fluorescent populations (due to proton counting error). See Figure S1 for gating hierarchy.

A nearly identical gating strategy was employed to detect changes in autofluorescence following treatment, with the only exception being that viable cells were not gated based on calcein fluorescence (as these cells were un-dyed) (Figure S2A). Cells that were untreated and undyed (sham control) showed no autofluorescence in the calcein or the PI channels. However, slight spreading along the PI axis was observed in cells treated with ultrasound and PCCAs. The percentage of cells in Q3 was subtracted from final sonoporation efficiencies for each condition.
