# Supplementary figures and images for: An evaluation of the sonoporation potential of low-boiling point phase-change ultrasound contrast agents in vitro
Source: J Ther Ultrasound. 2017 Jan 24;5:7. doi: 10.1186/s40349-017-0085-z (PMC5260003; doi:10.1186/s40349-017-0085-z)

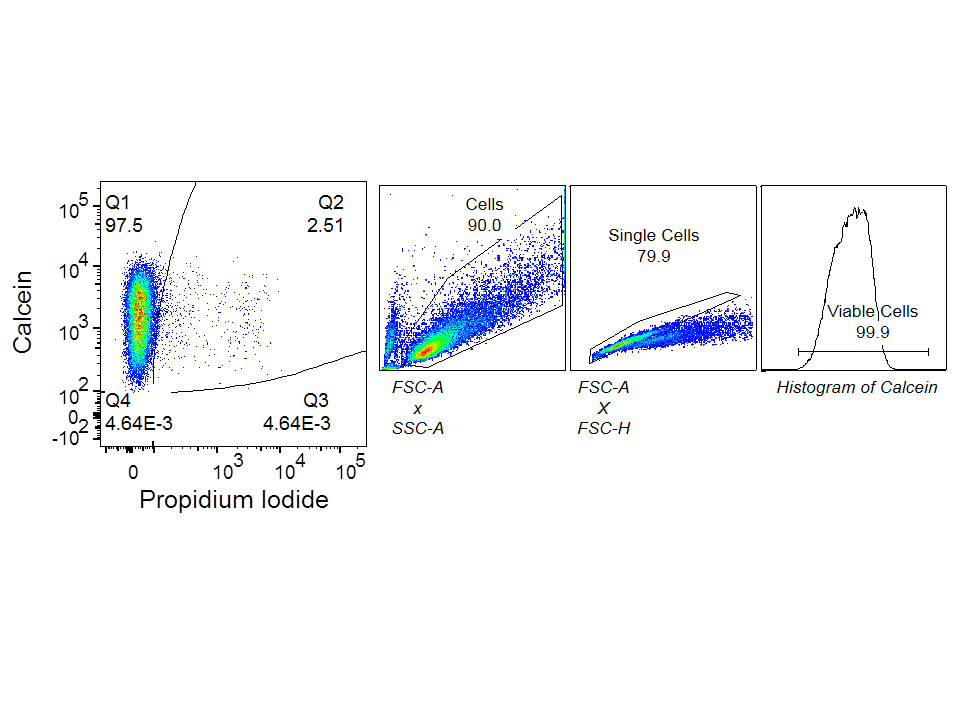

Supplement: Additional file 2: Figure S1. — Gating hierarchy used for sonoporation detection. (TIF 114 kb) [file 40349_2017_85_MOESM2_ESM.tif]

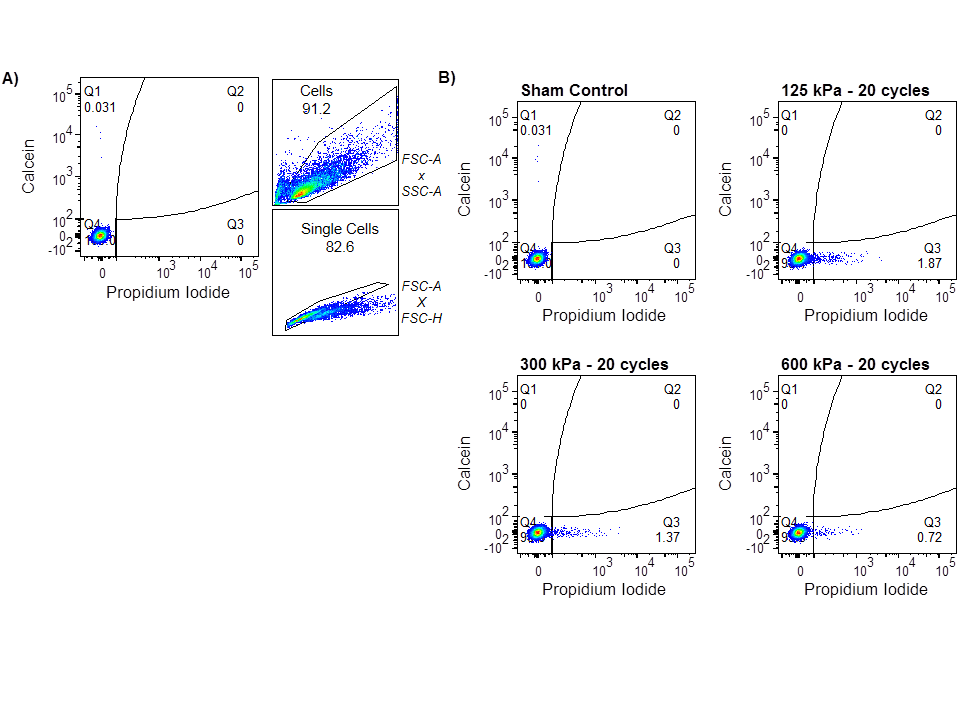

Supplement: Additional file 3: Figure S2. — A) Gating hierarchy for detecting autofluorescence in treated cells. First, cells were isolated from debris using FSC-A vs. SSC-A. Second, singlet cells were isolated using FSC-A vs. FSC-H. Third, quadrant gates were drawn identical to those used for quantifying sonoporation. B) Representative dot plots demonstrating slight spreading (autofluorescence) of cells treated with ultrasound and PCCAs. (TIF 123 kb) [file 40349_2017_85_MOESM3_ESM.tif]
